# Note to Dr. Horner’s Case of Luxation of the Cervical Vertebræ

**Published:** 1843-02-04

**Authors:** 


[The following note was intended to accompany Prof. Horner's case of luxation of the cervical vertebræ, published in the last number of the Examiner.]

Dr. Schuto, of Vienna, reported a case in the *Medizinische Jarhbuch des Osterrcichischen Staates*, for 1840, of a case of dislocation of the cervical vertebræ, which terminated favorably. A young man of twenty-four years, twisting suddenly his head, felt something give way in his neck, and the head remained immoveable. The next day his face was swollen, his head turned to the right, and bent towards the shoulder. His neck was arched slightly to the left side, but hollowed on the right. He complained of pain, which pressure on the third, fourth, and fifth cervical vertebræ increased. The head was perfectly immoveable; any attempt at motion was followed by great pain. The spinous processes of these vertebræ could not be traced. There was some feebleness of the right arm, and it required some effort to lift it. No other symptoms existed. An attempt at reduction, by lifting up the head, the trunk being fixed, failed. On the third day the numbness in the arm increased somewhat, and a further effort at reduction was made, and successfully. The patient being laid on the floor, the shoulders were fixed by folded sheets, a towel was passed under the chin, and the occiput was supported by both hands of an assistant: extension was then gradually made, until both patient and assistant experienced a snap, as of two bones meeting. On gradual relaxation of the extending force, the head was found in its natural position, and the power of motion was restored. On the next day there was rather more numbness, with some vertigo, and a quick pulse. Bleeding relieved all these unpleasant symptoms, and on the ninth day he left the hospital perfectly cured.—Ed. Med. Ex.

---

### A DRUNKEN POPULATION GETTING SHORTER.

In the department of Finisterre (Brittany,) the use of ardent spirits seems to increase, and to be attended with some peculiar effects on the population. In the two arrondissements of Quimper and Quimperlé, the spirituous liquors imported increased from 1,869 hectolitres in 1825, to 3,985 in 1839; and, corresponding with this increase, the average stature of young persons subject to military service is said to have diminished until it had become 23 millimetres (about an inch) less in 1838 than in 1818.

